# Reversal of rare paraneoplastic syndromes in melanoma: fever and cutaneous melanosis

**DOI:** 10.1097/CMR.0000000000001091

**Published:** 2026-03-09

**Authors:** Jasper J.L. van Geel, Ndidi J. Obihara, Dirk H. van Rens, Marye J. Boers-Sonderen, Johannes J. Bonenkamp, Kalijn F. Bol

**Affiliations:** a Department of Medical Oncology; b Department of Surgery, Radboud University Medical Center, Nijmegen, The Netherlands

**Keywords:** cutaneous melanosis, melanoma, paraneoplastic fever, paraneoplastic syndromes

## Abstract

Paraneoplastic syndromes (PNS), manifestations of systemic effects of a tumor, are rare in patients with melanoma. We describe two cases of patients who presented with rare PNS, fever, and cutaneous melanosis, that resolved after treatment of melanoma. The first patient, a 64-year-old woman, presented with high fever. After diagnostic evaluation, infectious causes were excluded and the patient was diagnosed with metastatic melanoma. The fever was attributed as a PNS. After surgical removal of a large necrotic intramuscular metastasis in her leg, her fever dissolved completely, allowing continuation of immunotherapy. The second patient, a 76-year-old man, presented with a grayish skin pigmentation and dark urine and referred to the gastroenterologist. He was diagnosed with metastatic melanoma and his symptoms were recognized as cutaneous melanosis. After initiation of immunotherapy, the pigmentation gradually faded. This case series show two distinct and rare paraneoplastic phenomena in melanoma. Early recognition and tailored management of PNS is crucial, as these syndromes may obscure underlying malignancy or influence patient’s overall condition. In our cases, PNS resolved after tumor-directed treatment.

## Introduction

Paraneoplastic syndromes (PNS) are symptoms that are a manifestation of systemic effects of a tumor, rather than by the local presence of tumor cells. They arise either from bioactive substances produced by the tumor, such as hormones and cytokines, or as part of an immune-mediated response triggered by the tumor. These syndromes can affect multiple organ systems, often correlate with high tumor burden, and may precede the diagnosis of the underlying malignancy.

While PNS is relatively common in certain tumor types, such as small cell lung cancer and renal cell cancer, PNS are rarely seen in patients with melanoma (<1%) [1–3]. PNS in patients with melanoma are mostly seen in the nervous system, skin, and as ophthalmologic manifestations [1].

This report describes two patients with metastatic melanoma who presented with distinct rare PNS, fever, and cutaneous melanosis, which were reversed by the treatment of the tumor.

Although neoplastic fever has been well described and documented in other tumor types, it is extremely rare in melanoma [4]. Paraneoplastic fever impacts the wellbeing of patients and can often be successfully suppressed with steroids. However, as immunotherapy is the preferred treatment for most patients with metastatic melanoma and steroids decrease the efficacy of immunotherapy, steroids are not desired and paraneoplastic fever can become a real challenge.

Cutaneous melanosis is another rare PNS, only described in patients with melanoma. Patients develop skin pigmentation because of melanin overproduction or dissemination, giving a grayish or ashen appearance. Melanosis has been associated with a poor prognosis [5].

## Case 1: fever

Our first case is a 64-year-old woman with a history of cutaneous melanoma (pT2aN1a, stage IIIA, BRAF mutant) treated with adjuvant pembrolizumab for 1 year.

**Fig. 1:**
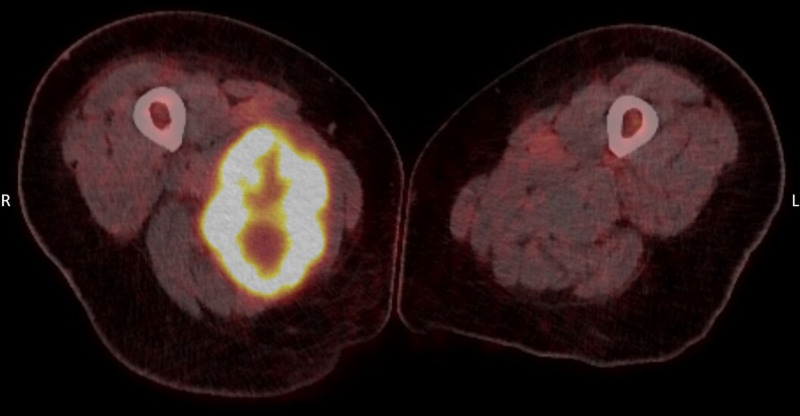
Fused FDG-PET/CT images showing a large, necrotic, FDG-avid metastasis in the upper right leg. The lesion has a largest diameter of 14.5 cm and is located near the abductor magnus muscle. CT, computed tomography; FDG, fluorodeoxyglucose.

Five years later, the patient was admitted with persistent high fever for 3 weeks, night sweats, progressive fatigue, and unintentional weight loss. Apart from the fever, there were no clinical signs of infection. Laboratory results showed white blood count of 13.3 × 10^9^/L and C-reactive protein of 81 mg/l. Chest radiography showed no pneumonia. Blood cultures, a respiratory panel, and HIV-testing were negative. Urine cultures grew an *Escherichia coli*. Empirical antibiotic treatment with piperacillin/tazobactam was initiated, without resolving the fever. An fluorodeoxyglucose PET/computed tomography (FDG PET/CT) scan showed a 14.5-cm large hypermetabolic necrotic intramuscular mass in the right upper leg (Fig. 1), a small lesion in the gluteal area on the left, and at least five FDG-avid subcutaneous lesions on the thoracic wall and right flank. The FDG PET/CT scan showed no signs of infection or other inflammatory foci. Histological biopsy of the thigh mass confirmed metastatic melanoma. Cerebral metastases were excluded with MRI.

Systemic therapy with ipilimumab (3 mg/kg) and nivolumab (1 mg/kg) was initiated. Despite this treatment, the patient continued to experience high fever, malaise, and severe fatigue and symptoms worsened, necessitating readmission to the hospital. There were no signs of immune-related toxicity. Repeat FDG PET/CT imaging showed no new infectious or inflammatory lesions that could explain the persistent fever. Therefore, the fever was interpreted as paraneoplastic fever secondary to metastatic melanoma.

Surgical resection of the large intramuscular lesion in the abductor compartment was performed. Histopathological examination confirmed metastatic melanoma with extensive tumor necrosis and no evidence of infection. A moderate amount of immune cells was observed in the tumor. Remarkably, the patient’s fever dissolved completely within a day after surgery and she started to improve clinically, supporting the diagnosis of paraneoplastic fever.

Postoperatively, the immunotherapy was continued. Follow-up PET/CT after four cycles of immunotherapy showed a mixed response of the known melanoma metastases. Patient was referred for palliative radiotherapy of two subcutaneous metastases. In addition, new, increased FDG subpleural uptake and FDG-avid hiliary lymph nodes were visible, most likely because of immunotherapy-induced pleuritis and a sarcoid-like reaction. Follow-up is ongoing without disease progression 6 months after start of immunotherapy.

## Case 2: cutaneous melanosis

**Fig. 2:**
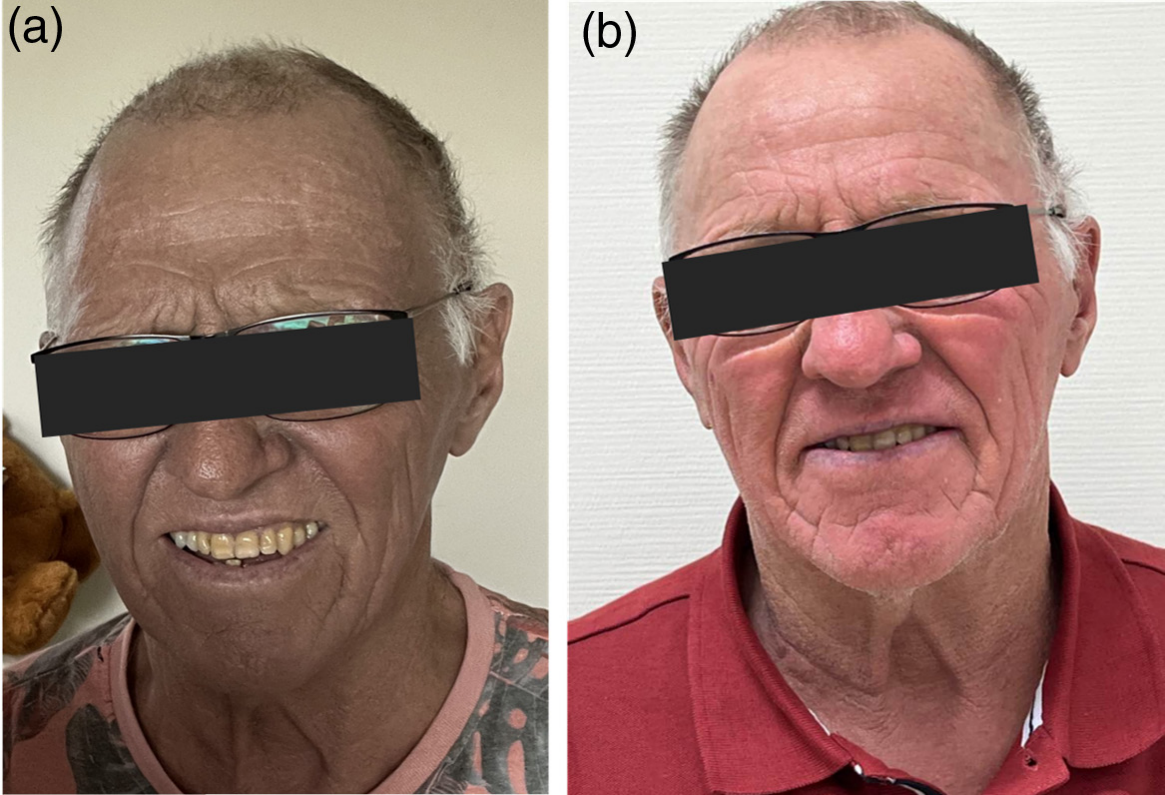
Patient with facial cutaneous melanosis. (a) Facial complexion prior to the start of immunotherapy. (b) Facial complexion 1 year after immunotherapy initiation.

A 76-year-old man presented with pain in the right flank, a dark, ashen facial complexion and brown urine. He was referred to a gastroenterologist under the suspicion of jaundice. However, laboratory tests revealed normal bilirubin levels, ruling out hepatic cholestasis. Further investigation led to the diagnosis of metastatic melanoma. The patient’s dark complexion was attributed to cutaneous melanosis, a rare paraneoplastic phenomenon (Fig. 2a).

The patient had a high tumor burden with numerous metastases in the liver, lungs, spleen, and brain, and an elevated lactate dehydrogenase (889 U/l). Molecular profiling showed *BRAF* wild-type status.

The patient commenced combination immunotherapy with ipilimumab (3 mg/kg) and nivolumab (1 mg/kg). After three cycles, the treatment was discontinued because of grade 3 immune-related colitis, managed with corticosteroids (prednisone 1 mg/kg) and infliximab (5 mg/kg + two times 10 mg/kg).

His elevated lactate dehydrogenase level normalized 2 months after treatment initiation, indicative of a response to immunotherapy. Radiological assessment 4 months after treatment initiation showed stable disease, according to the Response Evaluation Criteria in Solid Tumors criteria, with only minimal tumor regression.

Notably, the patient experienced a visible reduction of his ashen facial complexion approximately 6 months after treatment initiation, suggesting a slow reversal of the cutaneous melanosis upon response to immunotherapy (Fig. 2b).

Unfortunately, the patient showed progression of cerebral metastases, after previous reduction, during follow-up. The patient received stereotactic radiotherapy. Half a year later, he also showed extracerebral progression and immunotherapy was restarted. The cutaneous melanosis did not return upon tumor progression. He currently receives nivolumab monotherapy and CT scans show stable disease.

## Discussion

PNS associated with melanoma are rare and can mimic other clinical conditions, complicating timely and accurate diagnosis. Cutaneous melanosis and fever are even more rare subtypes of PNS. Their resolution following effective treatment underscores the importance of recognizing these syndromes early in the disease course.

Cutaneous melanosis, characterized by diffuse skin hyperpigmentation, may be misinterpreted as jaundice – particularly when accompanied by dark urine, as observed in our patient. Melanosis typically correlates with a high tumor burden and poor prognosis. According to the existing literature, life expectancy in such cases ranges from several weeks to a few months [5]. Given that immunotherapy often requires time to elicit a clinical response and may be less effective in patients with extensive disease, the presence of melanosis raises concerns about the therapeutic efficacy of immunotherapy [6]. For the first time, we demonstrates resolution of cutaneous melanosis following immune checkpoint inhibition in a patient with widespread metastatic melanoma. Notably, reversal of pigment deposition occurred gradually over several months.

While cutaneous melanosis is considered a poor prognostic indicator, another and more common skin-related PNS, vitiligo, represents a notable exception in melanoma. This depigmentation disorder arises from an autoimmune response in which cytotoxic T cells target melanocytes, both benign and malignant. In patients with metastatic melanoma, vitiligo can arise spontaneously (considered a PNS) or as an immune-related adverse event upon immunotherapy. The emergence of vitiligo – particularly during or after immunotherapy – has been associated with enhanced antitumor immunity and improved clinical outcomes [7]. Interestingly, even spontaneous vitiligo occurring before treatment has been linked to favorable prognosis [8], suggesting a preexisting immunological recognition of melanoma antigens.

The diagnostic process was more complex in the patient who presented with persistent fever. After exhaustive evaluation and an unsuccessful empirical treatment with antibiotics, the fever was ultimately attributed to a paraneoplastic origin. Paraneoplastic fever is a diagnosis of exclusion and is particularly rare in melanoma, where infectious etiologies are far more prevalent. It is thought to result from inflammatory cytokine release or tumor necrosis. In this case, the patient’s clinical status was significantly compromised by the fever and associated symptoms. Although corticosteroids are commonly used to manage paraneoplastic fever, their immunosuppressive effects were undesirable given the recent initiation of immunotherapy. Instead, surgical excision of the largest metastatic lesion led to resolution of the fever, supporting the hypothesis of tumor-driven systemic inflammation. This intervention improved the patient’s overall condition and enabled continuation of immunotherapy. Resection of the necrotic metastasis might contributed to the efficacy of immunotherapy, as the inflammatory state caused by the necrotic metastasis created an immunosuppressive microenvironment that may inhibit the efficacy of immunotherapy [9].

In conclusion, this case series highlights the diagnostic and therapeutic challenges posed by rare PNS in melanoma. While cutaneous melanosis and fever may initially obscure the underlying malignancy, their resolution following targeted interventions – immunotherapy and surgical debulking – demonstrates the potential for meaningful clinical recovery. Moreover, the contrasting prognostic implications of different PNS, such as the favorable association of vitiligo, underscore the complex interplay between tumor biology and host immune response. Early recognition and tailored management of PNS may enhance treatment outcomes in patients with advanced melanoma.

## Acknowledgements

Informed consent has been obtained from both patients granting permission for the publication of images as part of this work.

### Conflict of interest

K.F.B. has received honoraria (institutional) from BMS, Novartis, and MSD. For the remaining authors, there are no conflicts of interest.
